# Association between defibrillation-to-adrenaline interval and short-term outcomes in patients with out-of-hospital cardiac arrest and an initial shockable rhythm

**DOI:** 10.1016/j.resplu.2024.100651

**Published:** 2024-05-01

**Authors:** Shoji Kawakami, Yoshio Tahara, Teruo Noguchi, Satoshi Yasuda, Hidenobu Koga, Jun-ichiro Nishi, Naohiro Yonemoto, Hiroshi Nonogi, Takanori Ikeda

**Affiliations:** a Department of Cardiology, Aso Iizuka Hospital, Fukuoka, Japan; b Department of Cardiovascular Medicine, National Cerebral and Cardiovascular Center, Suita, Japan; c Department of Cardiovascular Medicine, Tohoku University Graduate School of Medicine, Sendai, Japan; d Clinical Research Support Office, Aso Iizuka Hospital, Fukuoka, Japan; e Department of Public Health, Juntendo University School of Medicine, Tokyo, Japan; f Faculty of Health Science, Osaka Aoyama University, Minoo, Japan; g Department of Cardiovascular Medicine, Toho University Faculty of Medicine, Tokyo, Japan

**Keywords:** Adrenaline, Defibrillation, Shockable rhythm, Cardiac arrest

## Abstract

**Aim:**

The optimal timing of adrenaline administration after defibrillation in patients with out-of-hospital cardiac arrest (OHCA) and an initial shockable rhythm is unknown. We investigated the association between the defibrillation-to-adrenaline interval and clinical outcomes.

**Methods:**

Between 2011 and 2020, we enrolled 1,259,960 patients with OHCA into a nationwide prospective population-based registry in Japan. After applying exclusion criteria, 20,905 patients with an initial shockable rhythm documented at emergency medical services (EMS) arrival who received adrenaline after defibrillation were eligible for this study. Multivariable logistic regression analysis was used to predict favourable short-term outcomes: prehospital return of spontaneous circulation (ROSC), 30-day survival, or a favourable neurological outcome (Cerebral Performance Category 1 or 2) at 30 days. Patients were categorised into 2-minute defibrillation-to-adrenaline intervals up to 18 min, or more than 18 min.

**Results:**

At 30 days, 1,618 patients (8%) had a favourable neurological outcome. The defibrillation-to-adrenaline interval in these patients was significantly shorter than in patients with an unfavourable neurological outcome [8 (5–12) vs 11 (7–16) minutes; P < 0.001]. The proportion of patients with prehospital ROSC, 30-day survival, or a favourable neurological outcome at 30 days decreased as the defibrillation-to-adrenaline interval increased (P < 0.001 for trend). Multivariable analysis revealed that a defibrillation-to-adrenaline interval of > 6 min was an independent predictor of worse prehospital ROSC, 30-day survival, or neurological outcome at 30 days when compared with an interval of 4–6 min.

**Conclusion:**

A longer defibrillation-to-adrenaline interval was significantly associated with worse short-term outcomes in patients with OHCA and an initial shockable rhythm.

## Introduction

For patients with out-of-hospital cardiac arrest (OHCA), in addition to prompt cardiopulmonary resuscitation (CPR), immediate treatment is dictated by the cardiac arrest rhythm.^1^, ^2^ In patients with a shockable rhythm such as ventricular fibrillation or pulseless ventricular tachycardia, prompt defibrillation is recommended, with adrenaline reserved for patients with a persistent shockable rhythm. In a previous study, a longer call-to-adrenaline interval was associated with decreased both return of spontaneous circulation (ROSC) and 30-day survival in patients with an initial non-shockable rhythm, but not in patients with an initial shockable rhythm^3^. However, the appropriate timing of adrenaline administration after the first defibrillation attempt has not been established. In particular, the 2020 American Heart Association (AHA) guidelines recommend adrenaline administration after the second defibrillation attempt whereas the 2021 European Resuscitation Council (ERC) guidelines recommend it after the third defibrillation attempt.^1^, ^2^ Determining the appropriate timing for adrenaline administration might contribute to improving the prognosis of patients with cardiac arrest and a shockable rhythm. To address this knowledge gap, we used the All-Japan Utstein Registry, a prospective nationwide registry of patients who have experienced OHCA in Japan, to examine the association between defibrillation-to-adrenaline interval and clinical outcomes in patients with OHCA and an initial shockable rhythm.

## Methods

### Study design and participants

**Fig. 1:**
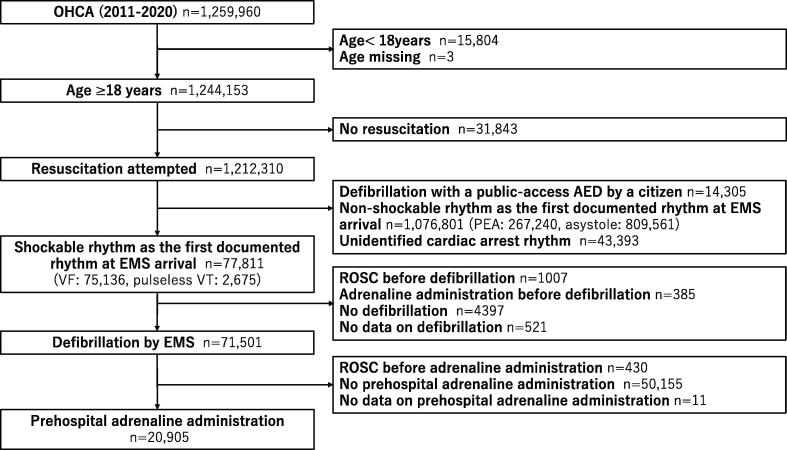
**Study flow chart.** OHCA, out-of-hospital cardiac arrest; AED, automated external defibrillator; PEA, pulseless electrical activity; EMS, emergency medical services; VF, ventricular fibrillation; VT, ventricular tachycardia; ROSC, return of spontaneous circulation.

This retrospective study analysed prospectively collected data in the All-Japan Utstein Registry of the Fire and Disaster Management Agency (FDMA), a prospective, population-based, nationwide registry of patients who have experienced OHCA,^4^, ^5^, ^6^, ^7^, ^8^ including 1,930,273 patients within the period from 2005 to 2020. We included adult OHCA patients who had an initial shockable rhythm at the time of emergency medical service (EMS) arrival, who received adrenaline after defibrillation by EMS, and who were transported to a medical institution from 2011 to 2020 (Fig. 1). Patients were excluded if any of the following criteria were met: under 18 years of age, resuscitation not attempted, a citizen performed defibrillation using a public access automated external defibrillator, initial non-shockable rhythm on EMS arrival, ROSC achieved or adrenaline administered before defibrillation, defibrillation not performed, ROSC achieved before adrenaline administration, or adrenaline not administered after defibrillation.

Data based on Utstein-style international guidelines for reporting OHCA were recorded.^9^, ^10^, ^11^ In brief, gender was recorded as biological male or female. Age, aetiology of arrest, bystander status, presence or absence of bystander CPR, presence or absence of dispatcher-assisted CPR, initial electrocardiogram rhythm, and treatments performed by EMS personnel were also recorded. The times of each of the following were recorded in real time by the EMS personnel on site: the call to the dispatcher, EMS arrival, defibrillation, adrenaline administration, and hospital arrival. The defibrillation-to-adrenaline interval was defined as the interval from the first defibrillation attempt to the first adrenaline administration by EMS. The primary outcome was a favourable neurological outcome, defined as a Cerebral Performance Category (CPC) score of 1 or 2 at 30 days. All survivors were followed for up to 30 days after OHCA. If the patient was discharged from the hospital before 30 days, the CPC score was recorded at the time of discharge. However, the database does not contain information regarding the date of hospital discharge.

The study was approved by the ethics committees of the National Cerebral and Cardiovascular Centre (R19040) and Aso Iizuka Hospital (R20076). The requirement of written informed consent from recruited patients was waived. A resuscitation science subcommittee of the Japanese Circulation Society was provided with the trial registry data after the prescribed governmental legal procedures were followed. We analysed only de-identified (anonymised) data. This study was registered with the University Hospital Medical Information Network Clinical Trials Registry (UMIN000009918).

### Field protocols for emergency medical services

Details on how the Japanese emergency system works have been described previously.^4^, ^5^, ^6^, ^7^, ^8^ In brief, FDMA manages a single emergency network with ambulance services that cover the entire country of Japan. All EMS personnel are trained to perform CPR according to Japan Resuscitation Council guidelines, which are based on the 2010 and 2015 International Liaison Committee on Resuscitation (ILCOR) guidelines.^3^, ^4^, ^5^, ^6^, ^7^, ^12^ In general, an ambulance crew consists of three EMS personnel, one of whom is an emergency lifesaving technician. With direct online medical direction, they administer adrenaline to patients aged > 8 years with pulseless electrical activity, ventricular fibrillation, or pulseless ventricular tachycardia rhythms after defibrillation, or with witnessed asystole.

### Statistical analysis

Statistical analysis was conducted using JMP version 16.2.0 (SAS Institute Japan, Tokyo, Japan). A P-value of < 0.05 was considered statistically significant. Data are expressed as medians [interquartile range (IQR)]. Intergroup comparisons of continuous variables were performed with the Wilcoxon rank-sum test. Nominal variables were compared using the χ^2^ test or Fisher’s exact test. Differences between the proportion of patients with a favourable short-term outcome (i.e., prehospital ROSC, 30-day survival, or favourable neurological outcome at 30 days) were analysed according to the defibrillation-to-adrenaline interval using the Cochran–Armitage test for trend. To examine whether variables predicted favourable short-term outcomes, multivariable logistic regression models were constructed using age, gender, year, district, presumed cardiac origin, witnessed arrest, bystander-initiated CPR, call-to-EMS arrival interval, EMS arrival-to-defibrillation interval, and defibrillation-to-adrenaline interval (Supplemental file S1).

## Results

### Study participants

During the study period, 1,259,960 patients were registered. After applying the exclusion criteria, 20,905 patients who had an initial shockable rhythm at EMS arrival and who received adrenaline after defibrillation were eligible (Fig. 1). Of these, 1,618 (8%) patients had a favourable neurological outcome and 19,287 (92%) did not. At 30 days, there were 1,215 (6%) patients with CPC 1; 403 (2%) patients with CPC 2; 627 (3%) patients with CPC 3; 1,393 (7%) patients with CPC 4; and 17,627 (83%) patients with CPC 5.

### Clinical characteristics and time intervals by favourable neurological outcome status

Table 1 shows comparisons of clinical characteristics and time management by favourable neurological outcome status. Each of the following intervals was significantly shorter in patients with a favourable neurological outcome than in those with an unfavourable neurological outcome: call to EMS arrival, EMS arrival to defibrillation, defibrillation to adrenaline, call to defibrillation, call to adrenaline, and call to hospital arrival.

**Table 1:** Baseline characteristics, prehospital characteristics, and time intervals in patients who received adrenaline in the prehospital setting by favourable neurological outcome status at 30 days.

	**Overall**	**Favourable neurological outcome**	**Unfavourable neurological outcome**	**Missing**	**P-value**
**Number**	20,905	1,618 (8)	19,287 (92)	0	
**Basic information**					
Age, years	69 (58–78)	60 (48–69)	70 (59–79)	0	<0.001
Male	16,807 (80)	785 (85)	10,603 (80)	0	<0.001
Year				0	0.395
2011	1,715 (8)	110 (6)	1,605 (94)		
2012	1,891 (9)	142 (8)	1,749 (92)		
2013	1,860 (9)	144 (8)	1,716 (92)		
2014	2,121 (10)	154 (7)	1,967 (93)		
2015	2,094 (10)	161 (8)	1,933 (92)		
2016	2,184 (10)	185 (8)	1,999 (92)		
2017	2,273 (11)	190 (8)	2,083 (92)		
2018	2,229 (11)	186 (8)	2,043 (92)		
2019	2,240 (11)	176 (8)	2,064 (92)		
2020	2,298 (11)	170 (7)	2,128 (93)		
District				0	<0.001
Hokkaido	1,121 (5)	83 (7)	1,038 (93)		
Tohoku	1,981 (9)	101 (5)	1,880 (95)		
Kanto	7,000 (33)	398 (6)	6,602 (94)		
Chubu	4,401 (21)	428 (10)	3,973 (90)		
Kinki	3,527 (17)	353 (10)	3,174 (90)		
Chugoku	838 (4)	58 (7)	780 (93)		
Shikoku	350 (2)	22 (6)	328 (94)		
Kyushu	1,687 (8)	175 (10)	1,512 (90)		
Presumed cardiac origin	19,041 (91)	1,556 (96)	17,485 (91)	0	<0.001
**Prehospital characteristic**					
Witnessed arrest	14,900 (71)	1,354 (84)	13,546 (70)	8	<0.001
by citizen	14,182 (68)	1,282 (9)	12,900 (91)		
by EMS responders	718 (3)	72 (10)	646 (90)		
Bystander-initiated CPR	11,153 (53)	967 (60)	10,186 (53)	0	<0.001
Chest compression-only CPR	10,033 (48)	861 (9)	9,172 (91)		
Conventional CPR with rescue breathing	1,119 (5)	106 (9)	1,013 (91)		
Unknown	1 (<1)	0 (0)	1 (1 0 0)		
Dispatcher-assisted CPR	12,132 (61)	970 (63)	11,162 (61)	976	0.176
Advanced airway management	12,964 (81)	864 (76)	12,100 (81)	4909	<0.001
Laryngeal mask airway	901 (6)	76 (8)	825 (92)		
Oesophageal obturator airway	10,069 (63)	669 (7)	9,400 (93)		
Endotracheal intubation	1,994 (12)	119 (6)	1,875 (94)		
Intravenous fluid	20,315 (97)	1,571 (97)	18,744 (97)	0	0.815
**Time course**					
Call-to-EMS arrival interval, minutes	7 (6–9)	7 (5–8)	7 (6–9)	14	<0.001
EMS arrival-to-defibrillation interval, minutes	3 (2–4)	3 (2–4)	3 (2–4)	74	<0.001
Defibrillation-to-adrenaline interval, minutes	11 (7–15)	8 (5–12)	11 (7–16)	151	<0.001
Call-to-defibrillation interval, minutes	11 (9–13)	9 (8–11)	11 (9–13)	53	<0.001
Call-to-adrenaline interval, minutes	22 (18–27)	18 (15–22)	22 (18–28)	132	<0.001
Call-to-hospital arrival interval, minutes	35 (29–43)	32 (27–39)	35 (29–43)	99	<0.001

Results are shown as medians (interquartile range) or number (%). CPR, cardiopulmonary resuscitation; EMS, emergency medical service.

**Fig. 2:**
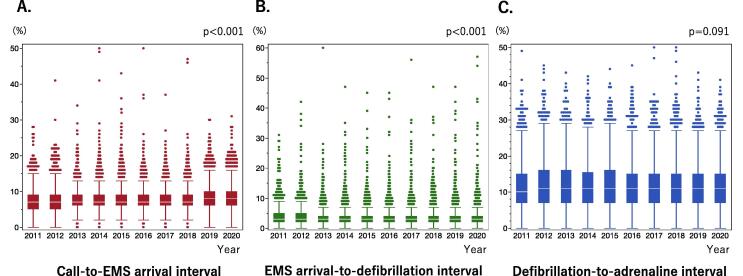
**Call-to-emergency medical services arrival interval (A), emergency medical service arrival-to-defibrillation interval (B), and defibrillation-to-adrenaline interval (C) by year (2011–2020).** EMS, emergency medical services.

Fig. 2 shows call-to-EMS arrival interval, EMS arrival-to-defibrillation interval, and defibrillation-to-adrenaline interval by year. There were no significant differences in defibrillation-to-adrenaline interval by year. Supplemental file S2 shows that there were no significant differences in the defibrillation-to-adrenaline interval between the periods from 2011 to 2015 and from 2016 to 2020 (P = 0.397).

### Associations between call-to-defibrillation, call-to-adrenaline, and defibrillation-to-adrenaline intervals and favourable short-term outcomes

**Fig. 3:**
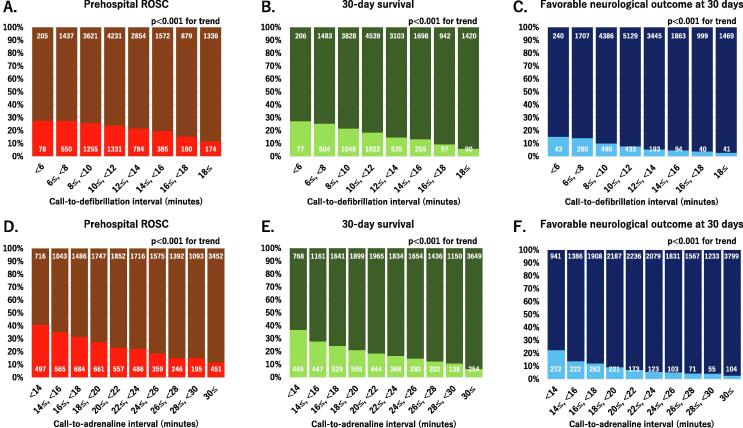
**Associations between the call-to-defibrillation interval and prehospital ROSC (A), 30-day survival (B), and a favourable neurological outcome at 30 days (C). Associations between the call-to-adrenaline interval and prehospital ROSC (E), 30-day survival (F), and favourable neurological outcome at 30 days (G).** Data are shown as percentages of all patients. The number in each bar indicates the number of patients. ROSC, return of spontaneous circulation.

Associations between the call-to-defibrillation or call-to-adrenaline interval and favourable short-term outcomes are shown in Fig. 3. As each time interval increased, the proportion of patients with favourable short-term outcomes significantly decreased.

**Fig. 4:**
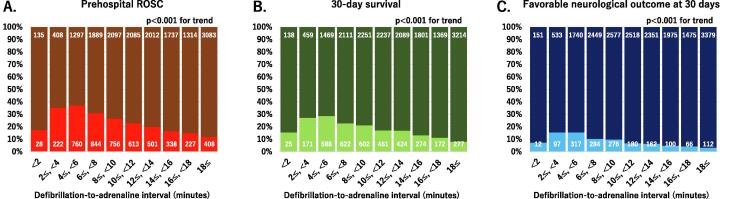
**Associations between the defibrillation-to-adrenaline interval and prehospital return of spontaneous circulation (A), 30-day survival (B), and a favourable neurological outcome at 30 days (C).** Data are shown as percentages of all patients. The number in each bar indicates the number of patients. ROSC, return of spontaneous circulation.

Associations between the defibrillation-to-adrenaline interval and favourable short-term outcomes are shown in Fig. 4. Patients with a defibrillation-to-adrenaline interval of < 2 min, ≥2 and < 4 min, or ≥ 4 and < 6 min comprised 0.8%, 3%, or 10% of patients, respectively. The proportion of patients with favourable short-term outcomes significantly increased as the defibrillation-to-adrenaline interval increased from < 2 min, to ≥ 2 and < 4 min, and then to ≥ 4 and < 6 min (P for trend < 0.05), and decreased when this interval was ≥ 6 min (P for trend < 0.001). A defibrillation-to-adrenaline interval of ≥ 4 and < 6 min was associated with the highest rates of patients with prehospital ROSC (37.0%), 30-day survival (28.6%), and a favourable neurological outcome (15.4%).

### Multivariable analysis for predicting favourable short-term outcomes

Table 2 and Supplemental files S3-S9 show the results of multivariable logistic analyses for predicting favourable short-term outcomes. With a defibrillation-to-adrenaline interval of ≥ 4 and < 6 min as a reference, the interval of ≥ 2 and < 4 min had the highest adjusted odds ratios for all short-term outcomes. Defibrillation-to-adrenaline intervals of < 2 min and ≥ 6 min were significant predictors for lack of prehospital ROSC, and the interval of ≥ 6 min was a significant predictor for < 30-day survival and a favourable neurological outcome at 30 days. A restricted cubic spline curve for the association between the defibrillation-to-adrenaline interval and a favourable neurological outcome at 30 days is shown in Supplemental file S10.

**Table 2:** Multivariable logistic regression analyses for predicting prehospital ROSC, 30-day survival, and favourable neurological outcome at 30 days, by the defibrillation-to-adrenaline interval in 2-minute intervals.

	**Prehospital ROSC** **(n = 20,706)**			**30-day survival** **(n = 20,706)**			**Favorable neurological outcome at 30 days** **(n = 20,706)**		
	Adjusted OR	95% CI	P-value	Adjusted OR	95% CI	P-value	Adjusted OR	95% CI	P-value
Defibrillation-to-adrenaline interval									
<2 min	0.53	0.35–0.82	0.004	0.77	0.48–1.23	0.268	0.70	0.37–1.33	0.271
≥2, <4 min	1.03	0.85–1.24	0.781	1.07	0.87–1.32	0.537	1.14	0.87–1.48	0.345
≥4, <6 min	Reference			Reference			Reference		
≥6, <8 min	0.75	0.67–0.85	<0.001	0.73	0.63–0.83	<0.001	0.63	0.53–0.76	<0.001
≥8, <10 min	0.61	0.54–0.69	<0.001	0.68	0.59–0.78	<0.001	0.61	0.51–0.73	<0.001
≥10, <12 min	0.49	0.43–0.56	<0.001	0.53	0.46–0.61	<0.001	0.42	0.34–0.51	<0.001
≥12, <14 min	0.42	0.37–0.49	<0.001	0.53	0.46–0.62	<0.001	0.41	0.33–0.50	<0.001
≥14, <16 min	0.33	0.29–0.39	<0.001	0.40	0.34–0.48	<0.001	0.30	0.24–0.38	<0.001
≥16, <18 min	0.29	0.25–0.35	<0.001	0.32	0.27–0.39	<0.001	0.26	0.20–0.35	<0.001
≥18 min	0.23	0.20–0.27	<0.001	0.24	0.20–0.28	<0.001	0.21	0.16–0.26	<0.001

The logistic regression model included the following variables: age, gender, year, district, origin of cardiac arrest, witnessed arrest, bystander-initiated cardiopulmonary resuscitation, call-to-EMS arrival interval, EMS arrival-to-defibrillation interval, and defibrillation-to-adrenaline interval. The results of analyses for variables other than the defibrillation-to-adrenaline interval are shown in Supplemental file S3. CI, confidence interval; EMS, emergency medical services; OR, odds ratio; ROSC, return of spontaneous circulation.

## Discussion

The present study analysed a nationwide, population-based, prospective registry, focusing on the time interval between defibrillation and adrenaline administration in the CPR algorithm for OHCA with an initial shockable rhythm. The main findings were as follows: (i) the defibrillation-to-adrenaline interval in patients with a favourable neurological outcome was significantly shorter than in patients with an unfavourable neurological outcome; (ii) the proportion of patients with favourable short-term outcomes, specifically prehospital ROSC, 30-day survival, and a favourable neurological outcome at 30 days, decreased as the time to adrenaline administration after defibrillation increased; and (iii) in multivariable analysis, the defibrillation-to-adrenaline interval independently predicted prehospital ROSC, 30-day survival, and a favourable neurological outcome at 30 days.

### Early adrenaline administration for patients with cardiac arrest

A systematic review and *meta*-analysis by ILCOR, which included the large, randomised, double-blind, placebo-controlled PARAMEDIC2 trial, found that intravenous adrenaline administration improved survival to hospital admission as well as long-term survival to 3 months, but it was unclear if it improved the likelihood of a favourable neurological outcome.^13^, ^14^ These data have led to international guidelines placing a very high value on the apparent life-preserving benefit of adrenaline, even if the absolute effect size is likely to be small and the effect on survival with a favourable neurological outcome is uncertain.^1^, ^2^ In a systematic review of 10 observational studies examining the timing of adrenaline administration, early adrenaline administration was associated with higher rates of ROSC,^15^ although early administration was variably defined as a call-to-adrenaline interval of < 5, <10, <15, 5–18, or 5–20 min, and most of these studies included both OHCA and in-hospital cardiac arrest patients, as well as shockable and non-shockable rhythm.^3^, ^12^, ^16^, ^17^, ^18^, ^19^ Moreover, the effects of adrenaline on ROSC relative to placebo were greater for patients with an initially non-shockable rhythm than those with a shockable rhythm.^20^ ILCOR recommends administering adrenaline as soon as feasible for non-shockable rhythms and after initial defibrillation attempts are unsuccessful during CPR for shockable rhythms.^1^, ^2^ In the present study, the association between a longer call-to-adrenaline interval and a lower proportion of patients with favourable short-term outcomes was consistent with the results of previous studies^12^, ^16^, ^19^.

### Importance of time management for adrenaline administration after defibrillation as a predictor of clinical outcomes

On the basis of the ILCOR recommendation, the AHA recommended adrenaline administration after the second defibrillation attempt^2^, while the ERC recommended it after the third defibrillation attempt in patients with a persistent shockable rhythm.^1^ If defibrillation is performed immediately after the rhythm check, adrenaline is initially administered 2 or 4 min after the first defibrillation. If a non-shockable rhythm develops after the first defibrillation, adrenaline administration is recommended as soon as possible. In the present study, when a defibrillation-to-adrenaline interval of ≥ 4 and < 6 min was used as a reference, the interval of ≥ 2 and < 4 had the highest adjusted odds ratios for all short-term outcomes, while the interval of ≥ 6 min was associated with significantly worse short-term outcomes. These results support the early administration of adrenaline in accordance with guideline recommendations.

On the other hand, use of adrenaline before defibrillation or within 2 min after the first defibrillation, which is contrary to these guidelines recommendations, was associated with worse survival outcomes for patients with in-hospital cardiac arrest due to a shockable rhythm.^21^, ^22^ There are several possible explanations for these findings: inappropriate use of adrenaline could lead to increased demand for myocardial oxygen and reduced blood flow to other organs for patients who might have achieved ROSC with defibrillation alone, or adrenaline administration might have contributed to delays in defibrillation.^21^, ^23^, ^24^, ^25^, ^26^ In the present study, when a defibrillation-to-adrenaline interval of < 2 min was used as a reference, the adjusted odds ratios for all favourable short-term outcomes were lower in patients in whom this interval was < 2 min than in those in whom it was ≥ 2 and < 4 or ≥ 4 and < 6 min. These results might reflect the disadvantages of premature adrenaline administration or the quality of guideline-based CPR.

### Study limitations

This study has several potential limitations. First, this was an observational study and it is subject to the weaknesses inherent to epidemiological studies (in terms of data integrity and validity). Although the general information bias that characterises epidemiological studies may also be present, there is no recall bias as the time indicators used in this study were recorded in real time by the EMS personnel at the scene. Second, this study represented only Japan, which may affect the generalizability of the findings. Third, data were unavailable regarding the electrocardiogram rhythm after the first defibrillation attempt, the quality of securing intravenous access for adrenaline, the quality of CPR, cerebral function after OHCA, and treatment strategies after hospital arrival. Fourth, since this study analysed OHCA patients with initial shockable rhythm, it cannot be generalised to patients with initial non-shockable rhythm. Fifth, this study did not examine the impact of the COVID-19 pandemic.

## Conclusion

A longer defibrillation-to-adrenaline interval is significantly associated with worse short-term outcomes in patients with OHCA and a shockable rhythm.

## CRediT authorship contribution statement

**Shoji Kawakami:** Writing – original draft, Visualization, Methodology, Investigation, Data curation, Conceptualization. **Yoshio Tahara:** Writing – review & editing, Supervision, Methodology, Investigation, Data curation, Conceptualization. **Teruo Noguchi:** Investigation, Conceptualization. **Satoshi Yasuda:** Writing – review & editing, Supervision, Conceptualization. **Hidenobu Koga:** Formal analysis, Data curation. **Jun-ichiro Nishi:** Investigation, Data curation. **Naohiro Yonemoto:** Formal analysis, Data curation. **Hiroshi Nonogi:** Writing – review & editing, Supervision, Conceptualization. **Takanori Ikeda:** Writing – review & editing, Supervision.

## Declaration of competing interest

The authors declare that they have no known competing financial interests or personal relationships that could have appeared to influence the work reported in this paper.
