# Supplementary material for: Association between defibrillation-to-adrenaline interval and short-term outcomes in patients with out-of-hospital cardiac arrest and an initial shockable rhythm
Source: Resusc Plus. 2024 May 1;18:100651. doi: 10.1016/j.resplu.2024.100651 (PMC11070920; doi:10.1016/j.resplu.2024.100651)
Supplement: Supplementary Data 1 [file mmc1.docx]

**Supplemental file S1: Directed acyclic graph of the relationship between the defibrillation-to-adrenaline interval and favourable neurological outcome at 30 days.** CPR, cardiopulmonary resuscitation; EMS, emergency medical service.

**Supplemental file S2: Comparison of the defibrillation-to-adrenaline interval in the periods from 2011 to 2015 and from 2016 to 2020.**

**Supplemental file S3. Multivariable logistic regression analyses for predicting prehospital ROSC, 30-day survival, and favourable neurological outcome at 30 days, by baseline characteristics**

|  | **Prehospital ROSC**  **(n=20,706)** | | | **30-day survival**  **(n=20,706)** | | | **Favourable neurological outcome at 30 days**  **(n=20,706)** | | |
| --- | --- | --- | --- | --- | --- | --- | --- | --- | --- |
|  | Adjusted OR | 95% CI | P-value | Adjusted OR | 95% CI | P-value | Adjusted OR | 95% CI | P-value |
| Age | 1.00 | 1.00-1.01 | <0.001 | 0.97 | 0.97-0.97 | <0.001 | 0.96 | 0.96-0.96 | <0.001 |
| Male | 0.86 | 0.79-0.94 | <0.001 | 0.94 | 0.85-1.04 | 0.265 | 0.98 | 0.84-1.13 | 0.774 |
| District |  |  |  |  |  |  |  |  |  |
| Hokkaido | 1.01 | 0.87-1.19 | 0.871 | 1.77 | 1.50-2.09 | <0.001 | 1.42 | 1.10-1.84 | 0.007 |
| Tohoku | 0.90 | 0.79-1.03 | 0.125 | 0.97 | 0.83-1.14 | 0.729 | 1.20 | 0.95-1.51 | 0.129 |
| Kanto | Reference | | | Reference | | | Reference | | |
| Chubu | 1.00 | 0.91-1.10 | 0.992 | 1.21 | 1.09-1.35 | <0.001 | 1.52 | 1.31-1.77 | <0.001 |
| Kinki | 0.97 | 0.88-1.08 | 0.598 | 1.45 | 1.30-1.61 | <0.001 | 1.63 | 1.39-1.91 | <0.001 |
| Chugoku | 0.99 | 0.82-1.19 | 0.881 | 1.46 | 1.19-1.79 | <0.001 | 1.64 | 1.22-2.22 | 0.001 |
| Shikoku | 0.80 | 0.59-1.08 | 0.147 | 0.91 | 0.64-1.30 | 0.613 | 1.46 | 0.91-2.32 | 0.114 |
| Kyushu | 1.03 | 0.90-1.17 | 0.688 | 1.52 | 1.31-1.75 | <0.001 | 2.02 | 1.66-2.47 | <0.001 |
| Year |  |  |  |  |  |  |  |  |  |
| 2011 | 1.01 | 0.87-1.19 | 0.854 | 0.71 | 0.59-0.85 | <0.001 | 0.75 | 0.57-0.97 | 0.030 |
| 2012 | 1.17 | 1.01-1.37 | 0.038 | 0.85 | 0.72-1.01 | 0.068 | 0.93 | 0.73-1.20 | 0.586 |
| 2013 | 1.20 | 1.03-1.40 | 0.017 | 0.93 | 0.78-1.10 | 0.391 | 1.04 | 0.82-1.33 | 0.729 |
| 2014 | 1.09 | 0.94-1.27 | 0.240 | 0.87 | 0.74-1.03 | 0.110 | 0.94 | 0.74-1.20 | 0.643 |
| 2015 | 1.05 | 0.91-1.22 | 0.518 | 0.90 | 0.77-1.07 | 0.236 | 1.05 | 0.83-1.33 | 0.667 |
| 2016 | 1.03 | 0.89-1.19 | 0.710 | 0.98 | 0.83-1.15 | 0.795 | 1.16 | 0.93-1.46 | 0.191 |
| 2017 | 1.13 | 0.98-1.30 | 0.105 | 0.96 | 0.82-1.30 | 0.649 | 1.11 | 0.89-1.40 | 0.348 |
| 2018 | 1.08 | 0.94-1.25 | 0.279 | 1.04 | 0.89-1.22 | 0.641 | 1.08 | 0.86-1.36 | 0.490 |
| 2019 | 1.07 | 0.93-1.24 | 0.350 | 1.07 | 0.92-1.26 | 0.383 | 1.04 | 0.82-1.30 | 0.767 |
| 2020 | Reference | | | Reference | | | Reference | | |
| Presumed cardiac origin | 0.97 | 0.86-1.09 | 0.597 | 1.85 | 1.56-2.20 | <0.001 | 2.14 | 1.64-2.80 | <0.001 |
| Witnessed arrest | 1.41 | 1.31-1.53 | <0.001 | 1.64 | 1.50-1.80 | <0.001 | 2.02 | 1.75-2.32 | <0.001 |
| Bystander-initiated CPR | 1.02 | 0.95-1.10 | 0.511 | 1.11 | 1.03-1.20 | 0.008 | 1.28 | 1.14-1.43 | <0.001 |
| Call-to-EMS arrival interval | 0.96 | 0.95-0.97 | <0.001 | 0.91 | 0.89-0.92 | <0.001 | 0.87 | 0.85-0.89 | <0.001 |
| EMS arrival-to-defibrillation interval | 0.92 | 0.90-0.93 | <0.001 | 0.90 | 0.88-0.92 | <0.001 | 0.90 | 0.87-0.92 | <0.001 |

The logistic regression model included the following variables: age, gender, year, district, origin of cardiac arrest, witnessed arrest, bystander-initiated cardiopulmonary resuscitation, call-to-EMS arrival interval, EMS arrival-to-defibrillation interval, and defibrillation-to-adrenaline interval. The results of the analysis for the defibrillation-to-adrenaline interval are shown in Table 2. CI, confidence interval; EMS, emergency medical services; OR, odds ratio; ROSC, return of spontaneous circulation.

**Supplemental file S4. Multivariable logistic regression analyses for predicting prehospital ROSC, 30-day survival, and favourable neurological outcome at 30 days, with a defibrillation-to-adrenaline interval of less than 2 minutes as a reference**

|  | **Prehospital ROSC**  **(n=20,706)** | | | **30-day survival**  **(n=20,706)** | | | **Favourable neurological outcome at 30 days**  **(n=20,706)** | | |
| --- | --- | --- | --- | --- | --- | --- | --- | --- | --- |
|  | Adjusted OR | 95% CI | P-value | Adjusted OR | 95% CI | P-value | Adjusted OR | 95% CI | P-value |
| Age | 1.00 | 1.00-1.01 | <0.001 | 0.97 | 0.97-0.97 | <0.001 | 0.96 | 0.96-0.96 | <0.001 |
| Male | 0.86 | 0.79-0.94 | <0.001 | 0.94 | 0.85-1.04 | 0.265 | 0.98 | 0.84-1.13 | 0.774 |
| District |  |  |  |  |  |  |  |  |  |
| Hokkaido | 1.01 | 0.87-1.19 | 0.871 | 1.77 | 1.50-2.09 | <0.001 | 1.42 | 1.10-1.84 | 0.007 |
| Tohoku | 0.90 | 0.79-1.03 | 0.125 | 0.97 | 0.83-1.14 | 0.729 | 1.20 | 0.95-1.51 | 0.129 |
| Kanto | Reference | | | Reference | | | Reference | | |
| Chubu | 1.00 | 0.91-1.10 | 0.992 | 1.21 | 1.09-1.35 | <0.001 | 1.52 | 1.31-1.77 | <0.001 |
| Kinki | 0.97 | 0.88-1.08 | 0.598 | 1.45 | 1.30-1.61 | <0.001 | 1.63 | 1.39-1.91 | <0.001 |
| Chugoku | 0.99 | 0.82-1.19 | 0.881 | 1.46 | 1.19-1.79 | <0.001 | 1.64 | 1.22-2.22 | 0.001 |
| Shikoku | 0.80 | 0.59-1.08 | 0.147 | 0.91 | 0.64-1.30 | 0.613 | 1.46 | 0.91-2.32 | 0.114 |
| Kyushu | 1.03 | 0.90-1.17 | 0.688 | 1.52 | 1.31-1.75 | <0.001 | 2.02 | 1.66-2.47 | <0.001 |
| Year |  |  |  |  |  |  |  |  |  |
| 2011 | 1.01 | 0.87-1.19 | 0.854 | 0.71 | 0.59-0.85 | <0.001 | 0.75 | 0.57-0.97 | 0.030 |
| 2012 | 1.17 | 1.01-1.37 | 0.038 | 0.85 | 0.72-1.01 | 0.068 | 0.93 | 0.73-1.20 | 0.586 |
| 2013 | 1.20 | 1.03-1.40 | 0.017 | 0.93 | 0.78-1.10 | 0.391 | 1.04 | 0.82-1.33 | 0.729 |
| 2014 | 1.09 | 0.94-1.27 | 0.240 | 0.87 | 0.74-1.03 | 0.110 | 0.94 | 0.74-1.20 | 0.643 |
| 2015 | 1.05 | 0.91-1.22 | 0.518 | 0.90 | 0.77-1.07 | 0.236 | 1.05 | 0.83-1.33 | 0.667 |
| 2016 | 1.03 | 0.89-1.19 | 0.710 | 0.98 | 0.83-1.15 | 0.795 | 1.16 | 0.93-1.46 | 0.191 |
| 2017 | 1.13 | 0.98-1.30 | 0.105 | 0.96 | 0.82-1.30 | 0.649 | 1.11 | 0.89-1.40 | 0.348 |
| 2018 | 1.08 | 0.94-1.25 | 0.279 | 1.04 | 0.89-1.22 | 0.641 | 1.08 | 0.86-1.36 | 0.490 |
| 2019 | 1.07 | 0.93-1.24 | 0.350 | 1.07 | 0.92-1.26 | 0.383 | 1.04 | 0.82-1.30 | 0.767 |
| 2020 | Reference | | | Reference | | | Reference | | |
| Presumed cardiac origin | 0.97 | 0.86-1.09 | 0.597 | 1.85 | 1.56-2.20 | <0.001 | 2.14 | 1.64-2.80 | <0.001 |
| Witnessed arrest | 1.41 | 1.31-1.53 | <0.001 | 1.64 | 1.50-1.80 | <0.001 | 2.02 | 1.75-2.32 | <0.001 |
| Bystander-initiated CPR | 1.02 | 0.95-1.10 | 0.511 | 1.11 | 1.03-1.20 | 0.008 | 1.28 | 1.14-1.43 | <0.001 |
| Call-to-EMS arrival interval | 0.96 | 0.95-0.97 | <0.001 | 0.91 | 0.89-0.92 | <0.001 | 0.87 | 0.85-0.89 | <0.001 |
| EMS arrival-to-defibrillation interval | 0.92 | 0.90-0.93 | <0.001 | 0.90 | 0.88-0.92 | <0.001 | 0.90 | 0.87-0.92 | <0.001 |
| Defibrillation-to-adrenaline interval |  |  |  |  |  |  |  |  |  |
| <2 minutes | Reference | | | Reference | | | Reference | | |
| ≥2, <4 minutes | 1.92 | 1.23-3.01 | 0.004 | 1.39 | 0.85-2.29 | 0.187 | 1.63 | 0.83-3.19 | 0.153 |
| ≥4, <6 minutes | 1.87 | 1.22-2.87 | 0.004 | 1.31 | 0.82-2.09 | 0.268 | 1.44 | 0.75-2.74 | 0.271 |
| ≥6, <8 minutes | 1.41 | 0.92-2.16 | 0.116 | 0.95 | 0.59-1.52 | 0.826 | 0.91 | 0.48-1.73 | 0.773 |
| ≥8, <10 minutes | 1.14 | 0.74-1.74 | 0.560 | 0.88 | 0.55-1.41 | 0.603 | 0.87 | 0.46-1.66 | 0.679 |
| ≥10, <12 minutes | 0.92 | 0.60-1.42 | 0.719 | 0.69 | 0.43-1.11 | 0.125 | 0.60 | 0.31-1.15 | 0.124 |
| ≥12, <14 minutes | 0.79 | 0.52-1.22 | 0.296 | 0.70 | 0.43-1.12 | 0.135 | 0.59 | 0.30-1.13 | 0.110 |
| ≥14, <16 minutes | 0.62 | 0.40-0.96 | 0.033 | 0.53 | 0.33-0.85 | 0.008 | 0.43 | 0.22-0.84 | 0.014 |
| ≥16, <18 minutes | 0.55 | 0.35-0.86 | 0.008 | 0.42 | 0.26-0.69 | <0.001 | 0.38 | 0.19-0.75 | 0.005 |
| ≥18 minutes | 0.44 | 0.28-0.67 | 0.002 | 0.31 | 0.19-0.50 | <0.001 | 0.30 | 0.15-0.58 | <0.001 |

CI, confidence interval; CPR, cardiopulmonary resuscitation; EMS, emergency medical services; OR, odds ratio; ROSC, return of spontaneous circulation.

**Supplemental file S5. Multivariable logistic regression analyses for predicting prehospital ROSC, 30-day survival, and favourable neurological outcome at 30 days, by defibrillation-to-adrenaline intervals under 18 minutes (by 1-minute increments) or over 18 minutes**

|  | **Prehospital ROSC**  **(n=20,706)** | | | **30-day survival**  **(n=20,706)** | | | **Favourable neurological outcome at 30 days**  **(n=20,706)** | | |
| --- | --- | --- | --- | --- | --- | --- | --- | --- | --- |
|  | Adjusted OR | 95% CI | P-value | Adjusted OR | 95% CI | P-value | Adjusted OR | 95% CI | P-value |
| Age | 1.00 | 1.00-1.01 | <0.001 | 0.97 | 0.97-0.97 | <0.001 | 0.96 | 0.96-0.96 | <0.001 |
| Male | 0.86 | 0.79-0.94 | <0.001 | 0.95 | 0.85-1.05 | 0.272 | 0.98 | 0.84-1.13 | 0.772 |
| District |  |  |  |  |  |  |  |  |  |
| Hokkaido | 1.01 | 0.87-1.19 | 0.872 | 1.77 | 1.50-2.09 | <0.001 | 1.42 | 1.10-1.84 | 0.007 |
| Tohoku | 0.90 | 0.79-1.03 | 0.122 | 0.97 | 0.83-1.14 | 0.745 | 1.20 | 0.95-1.51 | 0.127 |
| Kanto | Reference | | | Reference | | | Reference | | |
| Chubu | 1.00 | 0.91-1.10 | 0.971 | 1.21 | 1.09-1.35 | <0.001 | 1.52 | 1.30-1.77 | <0.001 |
| Kinki | 0.97 | 0.88-1.07 | 0.572 | 1.45 | 1.30-1.61 | <0.001 | 1.63 | 1.39-1.91 | <0.001 |
| Chugoku | 0.99 | 0.82-1.19 | 0.923 | 1.46 | 1.19-1.80 | <0.001 | 1.64 | 1.21-2.22 | 0.001 |
| Shikoku | 0.80 | 0.59-1.08 | 0.148 | 0.92 | 0.64-1.30 | 0.626 | 1.46 | 0.91-2.32 | 0.114 |
| Kyushu | 1.03 | 0.90-1.17 | 0.704 | 1.53 | 1.32-1.76 | <0.001 | 2.04 | 1.67-2.48 | <0.001 |
| Year |  |  |  |  |  |  |  |  |  |
| 2011 | 1.02 | 0.87-1.19 | 0.844 | 0.71 | 0.59-0.85 | <0.001 | 0.75 | 0.57-0.97 | 0.030 |
| 2012 | 1.18 | 1.01-1.37 | 0.037 | 0.85 | 0.72-1.01 | 0.065 | 0.93 | 0.73-1.19 | 0.561 |
| 2013 | 1.20 | 1.03-1.40 | 0.018 | 0.93 | 0.78-1.10 | 0.382 | 1.04 | 0.82-1.33 | 0.722 |
| 2014 | 1.09 | 0.94-1.27 | 0.241 | 0.87 | 0.74-1.03 | 0.101 | 0.94 | 0.74-1.20 | 0.637 |
| 2015 | 1.05 | 0.91-1.22 | 0.507 | 0.90 | 0.76-1.06 | 0.223 | 1.05 | 0.83-1.33 | 0.684 |
| 2016 | 1.03 | 0.89-1.19 | 0.681 | 0.98 | 0.83-1.15 | 0.765 | 1.16 | 0.93-1.46 | 0.190 |
| 2017 | 1.13 | 0.98-1.30 | 0.098 | 0.97 | 0.82-1.13 | 0.662 | 1.11 | 0.89-1.40 | 0.333 |
| 2018 | 1.09 | 0.94-1.26 | 0.251 | 1.04 | 0.89-1.22 | 0.633 | 1.09 | 0.86-1.36 | 0.478 |
| 2019 | 1.08 | 0.93-1.24 | 0.328 | 1.07 | 0.92-1.26 | 0.376 | 1.04 | 0.82-1.31 | 0.758 |
| 2020 | Reference | | | Reference | | | Reference | | |
| Presumed cardiac origin | 0.97 | 0.86-1.10 | 0.627 | 1.86 | 1.56-2.20 | <0.001 | 2.14 | 1.64-2.80 | <0.001 |
| Witnessed arrest | 1.41 | 1.30-1.52 | <0.001 | 1.64 | 1.49-1.79 | <0.001 | 2.02 | 1.75-2.32 | <0.001 |
| Bystander-initiated CPR | 1.02 | 0.95-1.10 | 0.525 | 1.11 | 1.03-1.20 | 0.009 | 1.28 | 1.14-1.43 | <0.001 |
| Call-to-EMS arrival interval | 0.96 | 0.95-0.97 | <0.001 | 0.91 | 0.89-0.92 | <0.001 | 0.87 | 0.85-0.89 | <0.001 |
| EMS arrival-to-defibrillation interval | 0.92 | 0.90-0.93 | <0.001 | 0.90 | 0.88-0.92 | <0.001 | 0.90 | 0.87-0.92 | <0.001 |
| Defibrillation-to-adrenaline interval |  |  |  |  |  |  |  |  |  |
| <1 minute | 0.28 | 0.13-0.64 | 0.002 | 0.71 | 0.33-1.53 | 0.383 | 1.03 | 0.41-2.58 | 0.952 |
| ≥1, <2 minutes | 0.65 | 0.39-1.10 | 0.108 | 0.78 | 0.43-1.43 | 0.427 | 0.49 | 0.20-1.20 | 0.118 |
| ≥2, <3 minutes | 0.94 | 0.68-1.29 | 0.696 | 1.17 | 0.82-1.67 | 0.390 | 1.14 | 0.73-1.77 | 0.570 |
| ≥3, <4 minutes | 0.95 | 0.74-1.22 | 0.687 | 1.00 | 0.76-1.32 | 0.999 | 1.07 | 0.76-1.51 | 0.696 |
| ≥4, <5 minutes | Reference | | | Reference | | | Reference | | |
| ≥5, <6 minutes | 0.87 | 0.72-1.05 | 0.139 | 0.98 | 0.80-1.20 | 0.814 | 0.94 | 0.73-1.21 | 0.617 |
| ≥6, <7 minutes | 0.69 | 0.58-0.83 | <0.001 | 0.76 | 0.62-0.94 | 0.010 | 0.60 | 0.46-0.79 | <0.001 |
| ≥7, <8 minutes | 0.69 | 0.58-0.83 | <0.001 | 0.67 | 0.55-0.83 | <0.001 | 0.62 | 0.47-0.81 | <0.001 |
| ≥8, <9 minutes | 0.61 | 0.51-0.74 | <0.001 | 0.75 | 0.62-0.92 | 0.006 | 0.70 | 0.54-0.90 | 0.006 |
| ≥9, <10 minutes | 0.51 | 0.42-0.61 | <0.001 | 0.58 | 0.47-0.72 | <0.001 | 0.47 | 0.35-0.62 | <0.001 |
| ≥10, <11 minutes | 0.48 | 0.40-0.58 | <0.001 | 0.58 | 0.47-0.72 | <0.001 | 0.44 | 0.33-0.59 | <0.001 |
| ≥11, <12 minutes | 0.43 | 0.35-0.52 | <0.001 | 0.46 | 0.37-0.57 | <0.001 | 0.36 | 0.26-0.49 | <0.001 |
| ≥12, <13 minutes | 0.38 | 0.31-0.46 | <0.001 | 0.51 | 0.41-0.64 | <0.001 | 0.40 | 0.30-0.54 | <0.001 |
| ≥13, <14 minutes | 0.40 | 0.33-0.49 | <0.001 | 0.54 | 0.43-0.68 | <0.001 | 0.38 | 0.28-0.53 | <0.001 |
| ≥14, <15 minutes | 0.31 | 0.25-0.39 | <0.001 | 0.46 | 0.36-0.58 | <0.001 | 0.30 | 0.21-0.42 | <0.001 |
| ≥15, <16 minutes | 0.30 | 0.24-0.37 | <0.001 | 0.33 | 0.26-0.43 | <0.001 | 0.28 | 0.19-0.40 | <0.001 |
| ≥16, <17 minutes | 0.28 | 0.22-0.36 | <0.001 | 0.35 | 0.27-0.46 | <0.001 | 0.29 | 0.20-0.42 | <0.001 |
| ≥17, <18 minutes | 0.25 | 0.20-0.33 | <0.001 | 0.28 | 0.21-0.38 | <0.001 | 0.21 | 0.14-0.33 | <0.001 |
| ≥18 minutes | 0.21 | 0.18-0.26 | <0.001 | 0.23 | 0.19-0.28 | <0.001 | 0.20 | 0.15-0.26 | <0.001 |

CI, confidence interval; CPR, cardiopulmonary resuscitation; EMS, emergency medical services; OR, odds ratio; ROSC, return of spontaneous circulation.

**Supplemental file S6. Baseline characteristics, prehospital characteristics, and time intervals in patients who received adrenaline in the prehospital setting by favourable neurological outcome status at 30 days (male stratification analysis)**

|  | **Overall** | **Favourable neurological outcome** | **Unfavourable neurological outcome** | **Missing** | **P-value** |
| --- | --- | --- | --- | --- | --- |
| **Number** | 16,807 | 1,365 (8) | 15,442 (92) | 0 |  |
| **Basic information** |  |  |  |  |  |
| Age, years | 69 (58–78) | 60 (48–69) | 70 (59–79) | 0 | <0.001 |
| Year |  |  |  | 0 | 0.559 |
| 2011 | 1,380 (8) | 92 (7) | 1,288 (93) |  |  |
| 2012 | 1,543 (9) | 120 (9) | 1,423 (92) |  |  |
| 2013 | 1,466 (9) | 127 (9) | 1,339 (91) |  |  |
| 2014 | 1,729 (10) | 132 (10) | 1,597 (92) |  |  |
| 2015 | 1,650 (10) | 132 (10) | 1,518 (92) |  |  |
| 2016 | 1,767 (11) | 155 (11) | 1,612 (91) |  |  |
| 2017 | 1,817 (11) | 161 (12) | 1,656 (91) |  |  |
| 2018 | 1,787 (11) | 148 (11) | 1,639 (92) |  |  |
| 2019 | 1,824 (11) | 150 (11) | 1,674 (92) |  |  |
| 2020 | 1,844 (11) | 148 (11) | 1,696 (92) |  |  |
| District |  |  |  | 0 | <0.001 |
| Hokkaido | 884 (5) | 67 (8) | 817 (92) |  |  |
| Tohoku | 1,568 (9) | 80 (5) | 1,488 (95) |  |  |
| Kanto | 5,671 (34) | 333 (6) | 5,338 (94) |  |  |
| Chubu | 3,569 (21) | 380 (11) | 3,189 (89) |  |  |
| Kinki | 2,828 (17) | 287 (10) | 2,541 (90) |  |  |
| Chugoku | 668 (4) | 45 (7) | 623 (93) |  |  |
| Shikoku | 274 (2) | 18 (7) | 256 (93) |  |  |
| Kyushu | 1,345 (8) | 155 (12) | 1,190 (88) |  |  |
| Presumed cardiac origin | 15,537 (92) | 1,316 (96) | 14,221 (92) | 0 | <0.001 |
| **Prehospital characteristic** |  |  |  |  |  |
| Witnessed arrest | 12,142 (72) | 1,147 (84) | 10,995 (71) | 4 | <0.001 |
| by citizen | 11,596 (69) | 1,092 (9) | 10,504 (91) |  |  |
| by EMS responders | 546 (3) | 55 (10) | 491 (90) |  |  |
| Bystander-initiated CPR | 8,902 (53) | 829 (61) | 8,073 (52) | 0 | <0.001 |
| Chest compression-only CPR | 8,027 (47) | 740 (9) | 7,287 (91) |  |  |
| Conventional CPR with rescue breathing | 874 (5) | 89 (10) | 785 (90) |  |  |
| Unknown | 1 (<1) | 0 (0) | 1 (100) |  |  |
| Dispatcher-assisted CPR | 9,627 (60) | 817 (62) | 8,810 (60) | 796 | 0.091 |
| Advanced airway management | 10,431 (81) | 736 (76) | 9,695 (81) | 3937 | <0.001 |
| Laryngeal mask airway | 729 (6) | 62 (9) | 667 (92) |  |  |
| Oesophageal obturator airway | 8,143 (63) | 582 (7) | 7,561 (93) |  |  |
| Endotracheal intubation | 1,559 (12) | 92 (6) | 1,467 (94) |  |  |
| Intravenous fluid | 16,359 (97) | 1,327 (97) | 15,032 (97) | 0 | 0.777 |
| **Time course** |  |  |  |  |  |
| Call-to-EMS arrival interval, minutes | 7 (6–9) | 7 (5–8) | 7 (6–9) | 14 | <0.001 |
| EMS arrival-to-defibrillation interval, minutes | 3 (2–4) | 2 (2–4) | 3 (2–4) | 63 | <0.001 |
| Defibrillation-to-adrenaline interval, minutes | 11 (7–15) | 8 (5–12) | 11 (7–15) | 113 | <0.001 |
| Call-to-defibrillation interval, minutes | 11 (9–13) | 9 (8–11) | 11 (9–13) | 44 | <0.001 |
| Call-to-adrenaline interval, minutes | 22 (18–27) | 18 (15–22) | 22 (18–28) | 96 | <0.001 |
| Call-to-hospital arrival interval, minutes | 35 (29–43) | 32 (27–39) | 35 (29–43) | 82 | <0.001 |

Results are shown as medians (interquartile range) or number (%). CPR, cardiopulmonary resuscitation; EMS, emergency medical service.

**Supplemental file S7. Multivariable logistic regression analyses for predicting prehospital ROSC, 30-day survival, and favourable neurological outcome at 30 days, by the defibrillation-to-adrenaline interval in 2-minute intervals (male stratification analysis)**

|  | **Prehospital ROSC**  **(n=16,655)** | | | **30-day survival**  **(n=16,655)** | | | **Favourable neurological outcome at 30 days**  **(n=16,655)** | | |
| --- | --- | --- | --- | --- | --- | --- | --- | --- | --- |
|  | Adjusted OR | 95% CI | P-value | Adjusted OR | 95% CI | P-value | Adjusted OR | 95% CI | P-value |
| Age | 1.00 | 1.00-1.01 | <0.001 | 0.97 | 0.97-0.98 | <0.001 | 0.96 | 0.96-0.96 | <0.001 |
| District |  |  |  |  |  |  |  |  |  |
| Hokkaido | 0.95 | 0.79-1.14 | 0.560 | 1.77 | 1.50-2.09 | <0.001 | 1.45 | 1.09-1.93 | 0.010 |
| Tohoku | 0.92 | 0.79-1.07 | 0.270 | 0.97 | 0.83-1.14 | 0.729 | 1.15 | 0.88-1.49 | 0.298 |
| Kanto | Reference | | | Reference | | | Reference | | |
| Chubu | 1.01 | 0.91-1.12 | 0.839 | 1.25 | 1.11-1.41 | <0.001 | 1.61 | 1.37-1.90 | <0.001 |
| Kinki | 0.96 | 0.86-1.07 | 0.486 | 1.49 | 1.32-1.68 | <0.001 | 1.61 | 1.35-1.91 | <0.001 |
| Chugoku | 0.92 | 0.86-1.14 | 0.433 | 1.39 | 1.11-1.75 | 0.005 | 1.51 | 1.07-2.12 | 0.018 |
| Shikoku | 0.71 | 0.50-1.02 | 0.062 | 1.03 | 0.70-1.50 | 0.884 | 1.41 | 0.84-2.36 | 0.196 |
| Kyushu | 1.01 | 0.96-1.29 | 0.148 | 1.62 | 1.39-1.90 | <0.001 | 2.19 | 1.77-2.71 | <0.001 |
| Year |  |  |  |  |  |  |  |  |  |
| 2011 | 0.92 | 0.77-1.10 | 0.358 | 0.70 | 0.58-0.86 | <0.001 | 0.73 | 0.55-0.97 | 0.031 |
| 2012 | 1.15 | 0.97-1.36 | 0.113 | 0.82 | 0.68-0.99 | 0.037 | 0.89 | 0.68-1.16 | 0.401 |
| 2013 | 1.17 | 0.98-1.38 | 0.079 | 0.92 | 0.76-1.11 | 0.378 | 1.05 | 0.81-1.37 | 0.707 |
| 2014 | 1.07 | 0.98-1.38 | 0.408 | 0.88 | 0.74-1.06 | 0.172 | 0.91 | 0.70-1.18 | 0.492 |
| 2015 | 1.08 | 0.91-1.27 | 0.390 | 0.89 | 0.74-1.06 | 0.191 | 0.98 | 0.76-1.27 | 0.893 |
| 2016 | 1.03 | 0.88-1.21 | 0.714 | 0.93 | 0.78-1.11 | 0.413 | 1.09 | 0.85-1.40 | 0.475 |
| 2017 | 1.12 | 0.95-1.31 | 0.184 | 0.94 | 0.79-1.12 | 0.512 | 1.08 | 0.85-1.38 | 0.539 |
| 2018 | 1.13 | 0.96-1.32 | 0.149 | 0.99 | 0.84-1.18 | 0.945 | 0.98 | 0.77-1.26 | 0.885 |
| 2019 | 1.04 | 0.88-1.22 | 0.642 | 1.06 | 0.90-1.26 | 0.485 | 1.00 | 0.78-1.28 | 0.994 |
| 2020 | Reference | | | Reference | | | Reference | | |
| Presumed cardiac origin | 0.95 | 0.82-1.12 | 0.520 | 1.71 | 1.40-2.08 | <0.001 | 1.85 | 1.36-2.50 | <0.001 |
| Witnessed arrest | 1.42 | 1.30-1.55 | <0.001 | 1.61 | 1.45-1.78 | <0.001 | 1.97 | 1.69-2.30 | <0.001 |
| Bystander-initiated CPR | 1.04 | 0.96-1.12 | 0.322 | 1.17 | 1.07-1.28 | <0.001 | 1.35 | 1.20-1.53 | <0.001 |
| Call-to-EMS arrival interval | 0.96 | 0.95-0.98 | <0.001 | 0.91 | 0.89-0.92 | <0.001 | 0.86 | 0.96-0.96 | <0.001 |
| EMS arrival-to-defibrillation interval | 0.91 | 0.89-0.93 | <0.001 | 0.90 | 0.88-0.92 | <0.001 | 0.89 | 0.86-0.92 | <0.001 |
| Defibrillation-to-adrenaline interval |  |  |  |  |  |  |  |  |  |
| <2 minutes | 0.48 | 0.29-0.80 | 0.005 | 0.81 | 0.48-1.36 | 0.421 | 0.91 | 0.47-1.77 | 0.787 |
| ≥2, <4 minutes | 0.99 | 0.80-1.23 | 0.932 | 1.03 | 0.81-1.30 | 0.821 | 1.11 | 0.83-1.48 | 0.494 |
| ≥4, <6 minutes | Reference | | | Reference | | | Reference | | |
| ≥6, <8 minutes | 0.75 | 0.65-0.86 | <0.001 | 0.72 | 0.62-0.84 | <0.001 | 0.64 | 0.53-0.78 | <0.001 |
| ≥8, <10 minutes | 0.57 | 0.50-0.66 | <0.001 | 0.67 | 0.58-0.78 | <0.001 | 0.63 | 0.51-0.76 | <0.001 |
| ≥10, <12 minutes | 0.48 | 0.42-0.56 | <0.001 | 0.54 | 0.46-0.63 | <0.001 | 0.42 | 0.34-0.52 | <0.001 |
| ≥12, <14 minutes | 0.44 | 0.38-0.51 | <0.001 | 0.54 | 0.46-0.64 | <0.001 | 0.41 | 0.33-0.52 | <0.001 |
| ≥14, <16 minutes | 0.31 | 0.26-0.36 | <0.001 | 0.40 | 0.33-0.47 | <0.001 | 0.30 | 0.23-0.39 | <0.001 |
| ≥16, <18 minutes | 0.28 | 0.23-0.33 | <0.001 | 0.30 | 0.25-0.38 | <0.001 | 0.27 | 0.20-0.37 | <0.001 |
| ≥18 minutes | 0.23 | 0.20-0.27 | <0.001 | 0.25 | 0.21-0.30 | <0.001 | 0.22 | 0.17-0.28 | <0.001 |

CI, confidence interval; CPR, cardiopulmonary resuscitation; EMS, emergency medical services; OR, odds ratio; ROSC, return of spontaneous circulation.

**Supplemental file S8. Baseline characteristics, prehospital characteristics, and time intervals in patients who received adrenaline in the prehospital setting by favourable neurological outcome status at 30 days (female stratification analysis)**

|  | **Overall** | **Favourable neurological outcome** | **Unfavourable neurological outcome** | **Missing** | **P-value** |
| --- | --- | --- | --- | --- | --- |
| **Number** | 4,098 | 253 (6) | 3,845 (94) | 0 |  |
| **Basic information** |  |  |  |  |  |
| Age, years | 76 (64–84) | 63 (50–72) | 76 (65–85) | 0 | <0.001 |
| Year |  |  |  | 0 | 0.376 |
| 2011 | 335 (8) | 18 (5) | 317 (95) |  |  |
| 2012 | 348 (8) | 22 (6) | 326 (94) |  |  |
| 2013 | 394 (10) | 17 (4) | 377 (96) |  |  |
| 2014 | 392 (10) | 22 (6) | 370 (94) |  |  |
| 2015 | 444 (11) | 29 (7) | 415 (93) |  |  |
| 2016 | 417 (10) | 30 (7) | 387 (93) |  |  |
| 2017 | 456 (11) | 29 (6) | 427 (94) |  |  |
| 2018 | 442 (11) | 38 (9) | 404 (91) |  |  |
| 2019 | 416 (10) | 26 (6) | 390 (94) |  |  |
| 2020 | 454 (11) | 22 (5) | 432 (95) |  |  |
| District |  |  |  | 0 | 0.009 |
| Hokkaido | 237 (6) | 16 (7) | 221 (93) |  |  |
| Tohoku | 413 (10) | 21 (5) | 392 (95) |  |  |
| Kanto | 1,329 (32) | 65 (5) | 1,264 (95) |  |  |
| Chubu | 832 (20) | 48 (6) | 784 (94) |  |  |
| Kinki | 699 (17) | 66 (9) | 633 (91) |  |  |
| Chugoku | 170 (4) | 13 (8) | 157 (92) |  |  |
| Shikoku | 76 (2) | 4 (5) | 72 (95) |  |  |
| Kyushu | 342 (8) | 20 (6) | 322 (94) |  |  |
| Presumed cardiac origin | 3,504 (86) | 240 (95) | 3,264 (85) | 0 | <0.001 |
| **Prehospital characteristic** |  |  |  |  |  |
| Witnessed arrest | 2,758 (67) | 207 (82) | 2,551 (66) | 4 | <0.001 |
| by citizen | 2,586 (63) | 190 (7) | 2,396 (93) |  |  |
| by EMS responders | 172 (4) | 17 (10) | 155 (90) |  |  |
| Bystander-initiated CPR | 2,251 (55) | 138 (55) | 2,113 (55) | 0 | 0.899 |
| Chest compression-only CPR | 2,006 (49) | 121 (6) | 1,885 (94) |  |  |
| Conventional CPR with rescue breathing | 245 (5) | 17 (7) | 228 (93) |  |  |
| Unknown | 0 (0) | 0 (0) | 0 (0) |  |  |
| Dispatcher-assisted CPR | 2,505 (64) | 153 (63) | 2,352 (64) | 180 | 0.881 |
| Advanced airway management | 2,533 (81) | 128 (77) | 2,405 (81) | 972 | 0.138 |
| Laryngeal mask airway | 172 (6) | 14 (8) | 158 (92) |  |  |
| Oesophageal obturator airway | 1,926 (62) | 87 (5) | 1,839 (95) |  |  |
| Endotracheal intubation | 435 (14) | 27 (6) | 408 (94) |  |  |
| Intravenous fluid | 3,956 (97) | 244 (96) | 3,712 (97) | 0 | 0.859 |
| **Time course** |  |  |  |  |  |
| Call-to-EMS arrival interval, minutes | 7 (6–9) | 7 (5–8) | 7 (6–10) | 0 | <0.001 |
| EMS arrival-to-defibrillation interval, minutes | 3 (2–4) | 3 (2–4) | 3 (2–4) | 11 | <0.001 |
| Defibrillation-to-adrenaline interval, minutes | 11 (7–16) | 8 (5–12) | 11 (7–16) | 38 | <0.001 |
| Call-to-defibrillation interval, minutes | 11 (9–13) | 10 (8–12) | 11 (9–13) | 9 | <0.001 |
| Call-to-adrenaline interval, minutes | 22 (18–28) | 18 (15–22) | 23 (18–28) | 36 | <0.001 |
| Call-to-hospital arrival interval, minutes | 36 (30–43) | 32 (28–39) | 36 (30–43) | 17 | <0.001 |

Results are shown as medians (interquartile range) or number (%). CPR, cardiopulmonary resuscitation; EMS, emergency medical service.

**Supplemental file S9. Multivariable logistic regression analyses for predicting prehospital ROSC, 30-day survival, and favourable neurological outcome at 30 days, by the defibrillation-to-adrenaline interval in 2-minute intervals (female stratification analysis)**

|  | **Prehospital ROSC**  **(n=4,051)** | | | **30-day survival**  **(n=4,051)** | | | **Favourable neurological outcome at 30 days**  **(n=4,051)** | | |
| --- | --- | --- | --- | --- | --- | --- | --- | --- | --- |
|  | Adjusted OR | 95% CI | P-value | Adjusted OR | 95% CI | P-value | Adjusted OR | 95% CI | P-value |
| Age | 0.99 | 0.99-1.00 | 0.007 | 0.96 | 0.96-0.97 | <0.001 | 0.96 | 0.95-0.96 | <0.001 |
| District |  |  |  |  |  |  |  |  |  |
| Hokkaido | 1.25 | 0.91-1.73 | 0.173 | 1.66 | 1.13-2.43 | 0.010 | 1.33 | 0.73-2.46 | 0.354 |
| Tohoku | 0.86 | 0.64-1.15 | 0.318 | 0.99 | 0.68-1.43 | 0.946 | 1.40 | 0.82-2.40 | 0.216 |
| Kanto | Reference | | | Reference | | | Reference | | |
| Chubu | 0.98 | 0.79-1.20 | 0.822 | 1.04 | 0.80-1.36 | 0.775 | 1.10 | 0.73-1.66 | 0.655 |
| Kinki | 1.01 | 0.81-1.25 | 0.949 | 1.26 | 0.97-1.64 | 0.088 | 1.80 | 1.23-2.64 | 0.003 |
| Chugoku | 1.24 | 0.84-1.83 | 0.276 | 1.76 | 1.10-2.83 | 0.019 | 2.38 | 1.22-4.65 | 0.011 |
| Shikoku | 1.11 | 0.61-1.99 | 0.738 | 0.45 | 0.16-1.27 | 0.131 | 1.72 | 0.59-5.07 | 0.324 |
| Kyushu | 0.77 | 0.56-1.04 | 0.090 | 1.09 | 0.75-1.56 | 0.658 | 1.21 | 0.70-2.09 | 0.501 |
| Year |  |  |  |  |  |  |  |  |  |
| 2011 | 1.44 | 1.03-2.02 | 0.034 | 0.77 | 0.49-1.19 | 0.236 | 0.92 | 0.46-1.83 | 0.811 |
| 2012 | 1.28 | 0.91-1.79 | 0.159 | 1.04 | 0.68-1.58 | 0.870 | 1.24 | 0.65-2.38 | 0.518 |
| 2013 | 1.36 | 0.98-1.89 | 0.062 | 1.01 | 0.67-1.53 | 0.967 | 1.02 | 0.52-2.01 | 0.953 |
| 2014 | 1.20 | 0.86-1.67 | 0.277 | 0.84 | 0.55-1.29 | 0.424 | 1.18 | 0.62-2.24 | 0.608 |
| 2015 | 0.98 | 0.71-1.36 | 0.914 | 0.99 | 0.66-1.48 | 0.957 | 1.51 | 0.83-2.75 | 0.179 |
| 2016 | 1.02 | 0.73-1.42 | 0.906 | 1.27 | 0.86-1.88 | 0.237 | 1.64 | 0.90-2.98 | 0.103 |
| 2017 | 1.17 | 0.86-1.61 | 0.321 | 1.06 | 0.72-1.57 | 0.768 | 1.34 | 0.74-2.45 | 0.335 |
| 2018 | 0.92 | 0.66-1.27 | 0.607 | 1.27 | 0.87-1.86 | 0.220 | 1.81 | 1.02-3.20 | 0.043 |
| 2019 | 1.19 | 0.86-1.64 | 0.292 | 1.10 | 0.74-1.65 | 0.631 | 1.27 | 0.68-2.35 | 0.457 |
| 2020 | Reference | | | Reference | | | Reference | | |
| Presumed cardiac origin | 1.02 | 0.81-1.27 | 0.895 | 2.38 | 1.69-3.33 | <0.001 | 3.34 | 1.85-6.01 | <0.001 |
| Witnessed arrest | 1.41 | 1.20-1.67 | <0.001 | 1.84 | 1.48-2.29 | <0.001 | 2.21 | 1.57-3.13 | <0.001 |
| Bystander-initiated CPR | 0.96 | 0.82-1.11 | 0.565 | 0.88 | 0.72-1.06 | 0.168 | 0.96 | 0.73-1.28 | 0.801 |
| Call-to-EMS arrival interval | 0.95 | 0.98-1.05 | <0.001 | 0.91 | 0.87-0.94 | <0.001 | 0.90 | 0.85-0.96 | <0.001 |
| EMS arrival-to-defibrillation interval | 0.94 | 0.91-0.96 | <0.001 | 0.91 | 0.87-0.95 | <0.001 | 0.93 | 0.88-0.99 | 0.013 |
| Defibrillation-to-adrenaline interval |  |  |  |  |  |  |  |  |  |
| <2 minutes | 0.72 | 0.32-1.62 | 0.426 | 0.61 | 0.19-1.98 | 0.412 | -* | -† | -‡ |
| ≥2, <4 minutes | 1.16 | 0.76-1.77 | 0.499 | 1.22 | 0.75-2.00 | 0.421 | 1.20 | 0.65-2.24 | 0.153 |
| ≥4, <6 minutes | Reference | | | Reference | | | Reference | | |
| ≥6, <8 minutes | 0.79 | 0.60-1.05 | 0.101 | 0.80 | 0.58-1.12 | 0.198 | 0.58 | 0.37-0.93 | 0.022 |
| ≥8, <10 minutes | 0.74 | 0.56-0.98 | 0.035 | 0.70 | 0.50-0.97 | 0.035 | 0.51 | 0.33-0.81 | 0.004 |
| ≥10, <12 minutes | 0.55 | 0.41-0.74 | <0.001 | 0.49 | 0.34-0.71 | <0.001 | 0.42 | 0.25-0.69 | <0.001 |
| ≥12, <14 minutes | 0.38 | 0.27-0.52 | <0.001 | 0.51 | 0.35-0.75 | <0.001 | 0.41 | 0.24-0.70 | 0.001 |
| ≥14, <16 minutes | 0.45 | 0.32-0.62 | <0.001 | 0.45 | 0.30-0.68 | <0.001 | 0.33 | 0.18-0.61 | <0.001 |
| ≥16, <18 minutes | 0.36 | 0.25-0.52 | <0.001 | 0.41 | 0.26-0.63 | <0.001 | 0.21 | 0.10-0.43 | <0.001 |
| ≥18 minutes | 0.25 | 0.19-0.34 | <0.001 | 0.19 | 0.12-0.28 | <0.001 | 0.16 | 0.09-0.29 | <0.001 |

*, †, ‡ The odds ratio, confidence interval and p-value for the category of defibrillation-to-adrenaline interval <2 minutes were not available for analysis because the number of patients with a favourable neurological outcome at 30 days was 0 and the number of patients with an unfavourable neurological outcome at 30 days was 37. CI, confidence interval; EMS, emergency medical services; OR, odds ratio; ROSC, return of spontaneous circulation.

**Supplemental file S10: Restricted cubic spline curve for the association between the defibrillation-to-adrenaline interval and favourable neurological outcome at 30 days.**
